# The abilities of improved schizophrenia patients to work and live independently in the community: a 10-year long-term outcome study from Mumbai, India

**DOI:** 10.1186/1744-859X-8-24

**Published:** 2009-10-13

**Authors:** Amresh Kumar Srivastava, Larry Stitt, Meghana Thakar, Nilesh Shah, Gurusamy Chinnasamy

**Affiliations:** 1 Mental Health Foundation of India (PRERANA Charitable Trust) and Silver Mind Hospital, Mumbai, Maharashtra, India; 2 Department of Epidemiology & Biostatistics, Schulich School of Medicine & Dentistry, The University of Western Ontario, London, Ontario, Canada; 3 LTMG Hospital, University of Mumbai, Mumbai, Maharashtra, India; 4 Research Office, Schulich School of Medicine & Dentistry, The University of Western Ontario, London, Ontario, Canada; 5 Current address: Department of Psychiatry, Schulich School of Medicine & Dentistry, The University of Western Ontario, London, Ontario, Canada

## Abstract

**Background:**

The outcome of first episode schizophrenia has several determinants. Socioecological factors, particularly living conditions, migration, community and culture, not only affect the level of risk but also the outcome. Mega cities around the world show a unique socioecological condition that has several challenges for mental health. The present study reports on the long-term status of patients with schizophrenia in such a mega city: Mumbai, India.

**Aim:**

This study aims to reveal the long-term outcome of patients suffering from schizophrenia with special reference to clinical symptoms and social functioning.

**Methods:**

The cohort for this study was drawn from a 10-year follow-up of first episode schizophrenia. Patients having completed 10 years of consistent treatment after first hospitalisation were assessed on psychopathological and recovery criteria. Clinical as well as social parameters of recovery were evaluated. Descriptive statistics with 95% confidence intervals are provided.

**Results:**

Of 200 patients recruited at the beginning of this study, 122 patients (61%) were present in the city of Mumbai at the end of 10-year follow-up study period. Among 122 available patients, 101 patients (50.5%) were included in the assessment at the end of 10-year follow-up study period, 6 patients (3.0%) were excluded from the study due to changed diagnosis, and 15 patients (7.5%) were excluded due to admission into long-term care facilities. This indicates that 107 out of 122 available patients (87.7%) were living in the community with their families. Out of 101 (50.5%) patients assessed at the end of 10 years, 61 patients (30.5%) showed improved recovery on the Clinical Global Impression Scale, 40 patients (20%) revealed no improvement in the recovery, 43 patients (72.9%) were able to live independently, and 24 patients (40%) were able to find employment.

**Conclusion:**

With 10 years of treatment, the recovery rate among schizophrenia patients in Mumbai was 30.5%. Among the patients, 87.7% of patients lived in the community, 72.9% of patients lived independently, and 40% of patients obtained employment. However, 60% of patients were unable to return to work, which highlights the need for continued monitoring and support to prevent the deterioration of health in these patients. It is likely that socioecological factors have played a role in this outcome.

## Introduction

The outcome of schizophrenia is highly variable and heterogeneous. Despite good treatments, the long-term outcome of schizophrenia continues to be disappointing [1]. Long-term studies continue to report poor social adjustment, severe functional impairment, and high socioeconomic dependence in early-onset schizophrenia [2] as well as adult-onset schizophrenia. There are several well known determinants of outcome including duration of illness, age of onset, family support, service availability, personality and genetic factors. It is not quite clear how clinical, social and cultural factors interact to influence the short-term and long-term outcome of schizophrenia following treatment. Mega cities present a complex and unique challenge in service development [3] and social situation, which are detrimental to mental health. Changing environment, urban stress, living conditions, housing, pollution, urban poverty, population density, high cost of living, high cost of services, isolation from families, overcrowding, slum dwellings and sanitation are unique challenges responsible for diversion of funding and budget leading to poor attention on mental health issues. Poor accessibility and availability of mental health services, underutilisation of services, and increased risk and severity of mental disorders also add complexity to the outcome of schizophrenia patients in mega cities. The social determinants of health have been well established [4]. However, a better understanding of the impact of these factors on outcome of schizophrenia is needed.

Life in Mumbai, India, the fifth most populated city in the world with 19.2 million people [5], is complex, with merits and constraints to its provisions of psychiatric care. The city has primary, secondary and tertiary level of services, near-adequate number of psychiatrists with structured service provisions from government owned institutions. In addition, the private sector constitutes a major force in health care, providing additional emergency psychiatric facilities. The city has radial access to the network of family physicians who utilise a high referral system. The system is functional, accessible, available and evolving. However, people also face complex socioeconomic issues. Identification, awareness and stigma of mental illness continue to obstruct diagnosis, early intervention, continued treatment, people's participation and mental health promotion. Several factors such as urban poverty, excessive travelling time, long distances, working families, nuclear families, lack of social security, loneliness, unemployment, temporary job status and burden of caregiving interfere with accessing available facilities. Resources and manpower in mental health, however, continue to be less than adequate. An urban-rural difference in the outcome of schizophrenia is not a new perspective [6]. Studies have reported a negative social outcome from urban communities [7]. It has been repeatedly demonstrated that patients with schizophrenia often 'drift' toward marginalization in cities. There is a high prevalence of psychosis amongst the immigrant population and it is higher in second-generation immigrants as well [8].

Despite remarkable advancement in treatments, patients suffering from schizophrenia often do not have satisfactory outcomes in the long run. High rates of suicide attempt, disability, loss of vocation and inability to adapt to expected social role are some of the central issues. A recent study of 13 years of follow-up of early onset schizophrenia reported acute schizophrenic symptoms in 22.2% of patients and depression symptoms in 30.8% of patients [9]. The same study revealed that 37% of patients had tried to commit suicide or had seriously thought about it, and 77.8% of the former patients were still in outpatient treatment. Among the patients, 48.1% were reported to live with their parents, 33.3% lived in assisted or semiassisted conditions, and 18.5% were working in the open market [9]. Patients suffering from schizophrenia are unable to utilise existing employment opportunities. Employers neither recruit recovered subjects nor generate jobs for them. Poor social functioning and impoverishment lead to non-compliance and relapse, which further impairs the level of outcome. It is believed that such non-disease factors are modifiable to enhance the outcome status in schizophrenia [10]. In mega cities, unique strategies are required to provide mental health care that focuses not only on symptom remission but also on compliance, prevention of relapse, productivity and social functions. The aim of the present work is to study a 10-year outcome status of patients suffering from schizophrenia with special reference to clinical symptoms and social functioning in the city of Mumbai, India.

## Methods

This naturalistic cross-sectional study was conducted between 1993 and 2007 in a non-governmental Psychiatric Treatment Centre at Silver Mind Hospital (licensed centre as per the Indian Mental Health Act 1987), Mumbai, India. Ethics permission for this study was obtained from the local independent research ethics board.

A total of 200 hospitalised first-episode schizophrenia patients were recruited for a 10-year follow-up study. After obtaining an appropriate consent, each patient along with a key relative, was screened for diagnosis. Selected patients were entered into the study. The patients who were available (n = 107) at the end point of 10 years were assessed for recovery using the Clinical Global Impression Scale (CGIS) [11]. Those patients who showed improved recovery (n = 61) were further reassessed using the Positive and Negative Syndrome Scale (PANSS) [12] and the Hamilton Depression Rating Scale (HDRS) [13] for psychopathology. Social functioning was assessed using the Global Assessment of Functioning (GAF) [14] and Quality of Life (QOL) [15] scales. Status of employment and the ability to live independently were assessed on a locally developed measurement scale of 1 to 5. In the status of employment scale, 1, 2, 3, 4, and 5 means complete dependence, desire to earn, attempted to earn with failure, attempted to earn with success, and obtained satisfactory employment, respectively. In the ability to live independently, 1 means never lived independently, 2 means occasionally lived independently, 3 means none of the items mentioned in scale 5 despite assistance from others, 4 means all of the items mentioned in scale 5 but with the assistance of relatives, and 5 means able to do daily activities, social functions, work routines and organisations without anyone's assistance. Cognitive function was assessed using the Bender-Gestalt (BG) test [16] and the Wechsler Memory Scale (WMS) [17]. Extrapyramidal symptoms were rated using the Abnormal Involuntary Movement Scale (AIMS) [18].

The following inclusion criteria were used: (i) confirmed diagnosis of schizophrenia as per the Diagnostic and Statistical Manual, fourth edition (DSM-IV) [14], (ii) completion of 10 years of treatment with consistent follow-up and high compliance, (iii) those who scored 1 or 2 on CGIS indicating much improved and improved recovery status, (iv) willingness to participate in the assessment, (v) informed consent, and (vi) availability of a key relative. The exclusion criteria used in this study include: (i) a history of significant substance abuse and alcoholism, (ii) significant head trauma or neurological disorders during the follow-up period, (iii) any significant medical condition interfering with social functioning, (iv) poor level of compliance and inconsistent treatment, and (v) changed diagnosis. The outcome criteria used in this work are shown in Table 1. Data collection was performed using semistructured proforma. The collected data were subjected to descriptive statistics at 95% confidence intervals to know the treatment outcome on schizophrenia patients.

**Table 1:** Operational outcome criteria used in the study

**Criteria**	**Normal values**	**Abnormal values**
CGIS	<2	≥3
HDRS	<14	≥14
Social function:		
GAF	≥80	<80
QOL	≥80	<80
Status of employment	≥3	0 to 2
Independent living	≥3	0 to 2
Cognitive function:		
BG	<90	≥90
WMS	≥90	<90

BG = Bender-Gestalt test; CGIS = Clinical Global Impression Scale; GAF = Global Assessment of Functioning; HDRS = Hamilton Depression Rating Scale; QOL = Quality of Life; WMS = Wechsler Memory Scale.

## Results

Out of 200 patients recruited at the beginning of this study, 122 patients (61%) were present in the city of Mumbai at the end of 10-year follow-up study period (Table 2). Among 122 available patients, 101 patients (50.5%) were included in the assessment at the end of 10-year follow-up study period, 6 patients (3.0%) were excluded from the study due to changed diagnosis, and 15 patients (7.5%) were excluded due to admission into long-term care facilities. This indicates that 107 out of 122 available patients (87.7%) were living in the community with their families. The remaining 78 patients (39%) out of initially enrolled 200 patients were not available for the assessment due to various reasons: 18 patients (9%) moved out of Mumbai, 24 patients (12%) switched to another care provider, 19 patients (9.5%) discontinued the study, and 17 patients (8.5%) were lost in the follow-up due to withdrawal of consent and poor compliance. Out of 101 (50.5%) patients assessed at the end of 10 years, 61 patients (30.5%) showed improved recovery, and 40 patients (20%) revealed no improvement in recovery on CGIS.

**Table 2:** Clinical status of schizophrenia patients at the end of 10-year follow-up study period

**Clinical status of schizophrenia patients**	**Patients, n (%)**	**95% Confidence intervals (%)**
Total patients recruited at the beginning of study	200	N/A
Total patients available at the end of 10-year study period	122 (61.0%)	54.2 to 68.1
Patients included for follow-up assessment	101 (50.5%)	41.8 to 59.6
Improved	61 (30.5%)	24.2 to 37.4
Not improved	40 (20.0%)	14.7 to 26.2
Patients excluded from follow-up assessment due to changed diagnosis	6 (3.0%)	1.1 to 6.4
Patients excluded from follow-up assessment due to admission in the long-term care	15 (7.5%)	4.3 to 12.1
Total patients not available at the end of 10-year study period for follow-up assessment	78 (39.0%)	33.1 to 45.1
Moved out of Mumbai	18 (9.0%)	5.4 to 13.9
Switched to another care provider	24 (12.0%)	7.8 to 17.3
Discontinued from the study	19 (9.5%)	5.8 to 14.4
Withdrawal of consent and poor compliance	17 (8.5%)	5.0 to 13.3

N/A = not applicable.

Among the 61 patients who showed improved recovery (Table 3), 43 patients (70.5%) were male and their mean age was 42 years; 18 patients (29.5%) were female and their mean age was 41.5 years. Out of these 61 patients, 43 patients (72.9%) were able to live independently and 24 patients (40%) resumed their employment. Psychopathology was unremarkable, QOL was not very high, GAF was moderately satisfactory, the level of depression was mild, and cognition was marginally impaired. Those patients who did not show excellent recovery were also able to live in community, within their families, lacking significant improvement in clinical as well as social functions. They were continuing treatment and did not require any prolonged stay in hospital or in long-term residential houses. These patients did not display any significant threat of violence or lack of self-care or risk to physical health. Their families were able to work with the distress and dysfunction expressed by the patients. The families did not have any financial support from governmental or non-governmental organisations. The entire responsibility for care giving, treatments, health and nutrition was fulfilled by the family members and relatives of the patients.

**Table 3:** Characteristics of schizophrenia patients who showed improved recovery at the end of 10-year follow-up study period (n = 61)

**Characteristics of schizophrenia patients**	**Mean or frequency (SD or %)**	**Range**	**95% Confidence intervals (%)**
Male gender	43.0 (70.5%)	-	57.4 to 81.5
Male age (years)	42.0 (7.1)	22 to 58	39.7 to 44.2
Female gender	18.0 (29.5%)	-	18.5 to 42.6
Female age (years)	41.5 (8.0)	28 to 55	37.5 to 45.5
PANSS	49.4 (8.2)	31 to 68	47.3 to 51.5
Positive symptoms	8.0 (3.9)	2 to 20	7.0 to 9.0
Negative symptoms	10.1 (7.5)	1 to 27	8.2 to 12.0
General psychopathology	31.0 (12.7)	6 to 57	27.7 to 34.3
HDRS	12.5 (5.3)	4 to 24	11.1 to 13.9
GAF	78.3 (12.2)	45 to 98	75.2 to 81.5
QOL	76.2 (11.5)	46 to 98	73.2 to 79.1
Status of employment (>3)	24.0 (40.0%)		27.6 to 53.5
Independent living (>3)	43.0 (72.9%)		59.7 to 83.6
BG	98.4 (12.8)	78 to 128	95.1 to 101.7
WMS	90.3 (12.2)	68 to 117	87.1 to 93.4

BG = Bender-Gestalt test; GAF = Global Assessment of Functioning; HDRS = Hamilton Depression Rating Scale; PANSS = Positive and Negative Syndrome Scale; QOL = Quality of Life; SD = Standard Deviation; WMS = Wechsler Memory Scale.

## Discussion

The present study has shown that the long-term outcome of schizophrenia in Mumbai is poor. Furthermore, those patients who showed good outcome continued to live with disease symptoms and various levels of dysfunction. It appears that recovery is not an 'either/or' concept, but involves varying degrees and heterogeneity [19]. A good level of recovery requires total social integration and complete symptom remission. It has been previously recommended that outcome needs to be measured in at least two dimensions, clinical and social, to provide a more complete picture of the ability to function [20].

This study raises two pertinent questions: why does the long-term outcome of schizophrenia continue to be poor, and why do patients who recover continue to live with symptoms, distress and dysfunction? Further, there are two main perspectives arising from the present study, one shows that the numerical rate of outcome is 30.5% but 27.1% of patients remain dependent on others for living and 60% of patients do not succeed in gainful employment. The second and more satisfying perspective is that majority of these patients (87.7%) are living in the community within their families; 40% are employed and 72.9% are living independently from the subgroup, which showed improvement. Even those patients who did not improve significantly were also able to maintain living in the community without causing any significant risk.

There have been remarkable developments in the diagnosis, treatment, and rehabilitation of patients with schizophrenia. A number of newer drugs and psychosocial treatments have been found to be effective [21,22]. However, it appears that these advancements are insufficient to make a substantial difference for patients suffering from schizophrenia in this population. Of the 200 patients initially enrolled in the study, 30.5% were shown to have experienced improved and much improved outcome from schizophrenia based on CGIS. Even if we assume that those who migrated out of Mumbai city were stabilised and those who discontinued treatment were not actively ill, this percentage does not exceed 49%. However, this recovery rate is within the range (16% to 75%) reported in other long-term studies (Table 4) [20-40]. These results suggest that the long-term outcome of schizophrenia is similar in all regions and cultures and has not changed significantly over time.

**Table 4:** Long-term outcome status of schizophrenia patients reported in the literature

Study duration (years)	Recovery rate in schizophrenia patients	Reference
>10	33%	[20]
>10	50%	[21]
>10	51%	[22]
>10	40%	[23]
5	62%	[24]
5	64%	[25]
5	42%	[26]
5	62%	[27]
>10	50%	[28]
>10	64%	[29]
>10	75%	[30]
>10	62.7%	[31]
5	55%	[32]
5	16%	[33]
>10	64%	[34]
5	36%	[35]
5	57%	[36]
5	45%	[37]
5	65%	[38]
5	22%	[39]
>10	23%	[40]

Although there is considerable literature suggesting geographical and cultural factors influence risk as well as recovery from schizophrenia, biological theories continue to be in the forefront, implying that schizophrenia is a disease of the brain. Environmental, family and cultural factors may possibly influence the course of the illness, its manifestation, psychopathology, relapse, compliance and severity but not the final outcome of treatment [41]. This needs to be explored further. Social determinants of mental health play a pivotal role in illness progression but perhaps not in causation and response to treatment. The patients in the present cohort began participation with their first episode of schizophrenia. Patients had access to multidisciplinary team management, a structured community program, consistent treatment with atypical antipsychotics for at least 3 to 4 years and were highly compliant with medication, regularly attending psychosocial rehabilitation programs. Despite aggressive management, the 10-year recovery rate did not exceed 30.5%. A more positive side of the study is that the majority of these patients (87.7%) were able to live in communities with their families without any significant danger or risk. Only 7.5% of patients needed long-term supervised care. The Determinants of Outcome of Severe Mental Disorder (DOSMED) study of the World Health Organization also highlighted 'uniformity across cultures'. An international pilot study of schizophrenia carried out in 13 centres across the world and the DOSMED study showed that short-term outcome was more favourable in developing countries than in industrialised nations [42]. A large study of 18 cohorts reassessing long-term outcome also found heterogeneity in favourable outcome rates [28]. Unfortunately, an explicit definition of 'favourable' is not given, but appears to change with changes in social roles and medical advancements. 'Favourable' is a term which has carried on from the era when there were few advancements in pharmacotherapy or psychosocial management, and 'institutionalisation' was the sad reality in mental health. Parallel to physical health, changing expectations in mental health have been demanding.

It has also been argued that acute transient psychosis, a distinct feature of schizophrenia in India, is mostly responsible for better outcome as suggested in a 12-year follow-up study, which supported the International Classification of Diseases (10th revision) concept of a separable group of acute and transient psychotic disorders [42]. It therefore becomes clear that the short-term course of schizophrenia is reported to be better in some developing country settings. The long-term course of the disease in such settings, however, is not so clear. The DOSMED study at 2-year and 15-year follow-ups involving a cohort of first-contact patients in urban and rural Chandigarh, India showed that 92% of patients with a poor 2-year course had a poor long-term course and 47% died, a mortality rate nine times higher than patients with other 2-year course types [43]. The Madras longitudinal study with 76 patients followed for 10 years revealed that the clinical outcome was good in nearly 75% of the patients, with almost all symptoms showing a steep decline by the end of 10 years. In all, 59 subjects were asymptomatic at the end of the follow-up period and 12 were ill during the entire 10th year [44]. When compared to previously published findings, the present study shows a long-term good outcome rate of only 30.5%. This might be due to differences in the sociocultural milieu. Further studies are needed to provide more insights.

Another important aspect of the present study is the sociocultural milieu. The study was conducted in the world's fifth most populated mega city. Traditionally, patients in developing countries have shown very good outcomes in terms of clinical remission, less time spent in psychosis, and lower relapse rates [45]. There is very rich literature from India about the course, outcome and psychopathology, arguing that schizophrenia is transient, acute and more responsive with subjects being more integrated in their families. Studies suggest that families make a difference and contribute to good outcomes in developing countries, particularly in India, as compared to developed countries [46-49]. Recently, this premise has been challenged on the basis of natural selection bias, highlighting that the outcome of schizophrenia is not as good as previously projected [30]. It seems clear (and paradoxically so) that the course and outcome of schizophrenia in developing countries is deteriorating and getting closer to what is observed in the industrialised nations [50,51].

The present study further highlights the need to review outcome measures. When recovered patients were reassessed on the CGIS on multidimensional criteria of symptomatology, social function and employment, it was seen that 72.9% of patients were able to live independently and 40% resumed work. Independent living is a suggested criterion for outcome on multidimensional parameters [52]. This rate is higher than the 10-year follow-up rate of 56.8% reported in patients with schizophrenia comorbid with substance abuse in North America [53]. Independent living does indicate the individual's capacity for managing his/her life as well as being able to take care of their family. It suggests that all 'excellently improved' patients are unable to take control of their lives indicating a continued need for the involvement of caregivers, monitoring and support. It is not possible to say that after withdrawing support or monitoring whether these subjects would relapse or deteriorate. Much has been said about the 'return to function' of a person suffering from schizophrenia [31]. The traditional impression has been that of severe disability. In 2007, a Swedish study involving 5 years of follow-up treatment with antipsychotics reported that only 12% of the patients studied or worked full time [54]. However, new treatment modalities have made a difference. It certainly appears from the current study that patients in India recover better, showing that a sizable number (40%) have gained successful employment. A Chinese study reported that, at 10-year follow-up, 54% of patients with schizophrenia were able to work [55]. This is a definite improvement from rates reported two decades earlier. Recovered patients are able to take social roles and responsibilities. In the present study, it is unclear whether the 60% of patients who did not return to work were unemployable or victims of the stigma associated with mental illness that often leads to prejudice, discrimination and lack of opportunities. Both issues need to be addressed. The ability of these patients to work needs to be assessed as outcome criteria and suitable employment opportunities need to emerge [56].

The present study demonstrates relatively lower rates of clinical outcome, implying that schizophrenia may be a predominantly biological illness with a uniform recovery rate across cultures and regions. It shows limited social improvement in the patients, although a reasonable number return to gainful employment.

## Conclusion

The 10-year long-term outcome was studied in schizophrenia patients in Mumbai, India. The recovery rate among these patients was 30.5%. However, only a small fraction (7.5%) needed long-term supervised residential care. The majority of patients (87.7%) were able to live in the community. Fairly significant numbers of these patients (72.9%) lived independently and 40% of patients had obtained gainful employment. All recovered patients were not able to take control of their life. Although it is satisfying that a sizable number of patients returned to employment, there is a clear need for continued monitoring and support to prevent further decline and to maintain the level of recovery. Further studies are required to assess the causes for the low recovery rate in long-term outcome of schizophrenia.

## Competing interests

The authors declare that they have no competing interests.

## Authors' contributions

AS conceptualised, designed, supervised and wrote the study. LS statistically analysed the data and wrote the paper. MT conducted psychological and clinical assessments, regular follow-up, and data entry. NS reviewed study progress, interpreted data and wrote the paper. GC interpreted data, and reviewed, wrote and formatted the paper. All authors read and approved the final manuscript.
